# A Review of Desmopressin Use in Bleeding Disorders: An Unsung Hero?

**DOI:** 10.3390/biom15070967

**Published:** 2025-07-05

**Authors:** Benjamin Reardon, Leonardo Pasalic, Emmanuel J. Favaloro

**Affiliations:** 1 Joint Medical Program, School of Medicine and Public Health, University of Newcastle, Callaghan, NSW 2145, Australia; benjamin.reardon@health.nsw.gov.au; 2 Haematology Department, Calvary Mater Hospital Newcastle, Waratah, NSW 2298, Australia; 3 Haematology Department, Institute of Clinical Pathology and Medical Research (ICPMR), NSW Health Pathology, Westmead Hospital, Westmead, NSW 2145, Australia; leonardo.pasalic@health.nsw.gov.au; 4 Westmead Clinical School, University of Sydney, Westmead, NSW 2145, Australia; 5 Sydney Centres for Thrombosis and Haemostasis, Research and Education Network (REN) and Institute of Clinical Pathology and Medical Research (ICPMR), Westmead Hospital, Westmead, NSW 2145, Australia; 6 School of Dentistry and Medical Sciences, Faculty of Science and Health, Charles Sturt University, Wagga Wagga, NSW 2650, Australia; 7 School of Medical Sciences, Faculty of Medicine and Health, University of Sydney, Westmead Hospital, Westmead, NSW 2145, Australia

**Keywords:** desmopressin, DDAVP, hemophilia, von Willebrand disease, hemostasis

## Abstract

As a synthetic analogue of vasopressin, desmopressin or DDAVP has well established hemostatic properties. We present a review of DDAVP and summarize the clinical and laboratory evidence for its use in hemophilia A, von Willebrand disease (VWD), platelet function disorders, uremia, liver cirrhosis, and pregnancy, followed by illustrative examples of its broad efficacy from our local practice. In brief, DDAVP acts to release von Willebrand factor (VWF) and factor VIII from endogenously stored reserves, thereby correcting plasma deficiencies present in mild to moderately affected patients with hemophilia A and VWD. Thus, DDAVP represents a non-transfusional therapy for these disorders. Typically, a trial of DDAVP is arranged to assess individual responsiveness before employing DDAVP clinically, since there is individual variation in responsiveness. Thereafter, DDAVP can be utilized in responsive patients for clinical use and provides a factor replacement sparing strategy in these patients for some clinical situations. Nevertheless, DDAVP is best applied as a factor replacement sparing strategy, especially for minor procedures or short-term use.

## 1. Introduction and Principles of Desmopressin Use

**Figure 1:**
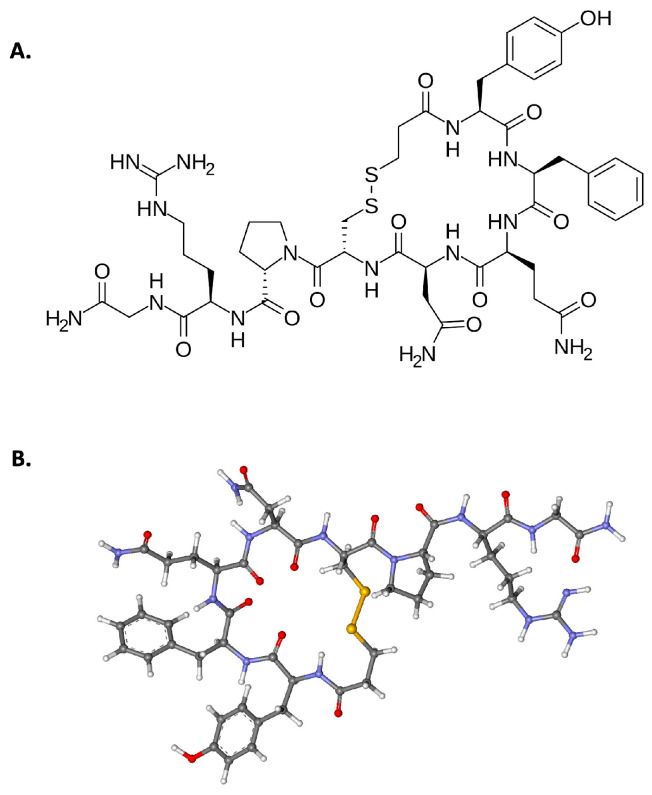
(**A**). Skeletal formula of desmopressin (1-deamino-8-D-arginine vasopressin, or DDAVP). Available at: https://en.wikipedia.org/wiki/Desmopressin#/media/File:Desmopressin.svg (accessed on 26 May 2025). (**B**). Ball-and-stick model of the desmopressin molecule. By Marina Vladivostok. Available at: https://en.wikipedia.org/wiki/Desmopressin#/media/File:Desmopressin_ball-and-stick.png (accessed on 26 May 2025). Both are reproduced under public domain/creative common attributions.

Desmopressin, DDAVP or 1-deamino-8-D-arginine, is a synthetic analogue of vasopressin that induces release of factor VIII (FVIII) and von Willebrand factor (VWF) from endothelial cells and platelet granules [1,2]. The structure of this biologically active biomolecule is given in Figure 1. Unlike the natural antidiuretic hormone, vasopressin, DDAVP does not produce vasoconstriction or increased blood pressure [3]. DDAVP was first used in management and prevention of bleeding patients with hemophilia A (HA) and von Willebrand disease (VWD) in the 1970s in the absence of additional blood products [4,5]. Following its use in these disorders, DDAVP use was expanded to congenital and acquired defects in platelet function, as well as abnormalities in hemostasis associated with chronic renal and hepatic disease [2].

HA is a common X-linked recessive disorder defined by a reduced FVIII activity to <40% [6]. In some cases, HA may be acquired through the development of FVIII inhibitors by various mechanisms [7]. In contrast, VWD is characterized by a defect and/or deficiency of VWF, sometimes also accompanied by a deficiency of FVIII; VWD is the most common inherited bleeding disorder worldwide [8,9,10]. HA is the second most common bleeding disorder but may represent the most common bleeding disorder in males. The prevalence of clinically significant VWD and HA are both close to 1 in 10,000 individuals (or 100/million of population) [11]. The main goal of treatment in patients with bleeding disorders, including both HA and VWD, is to prevent or lessen severity of bleeding, with broad therapeutic aims to increase plasma FVIII levels in HA, or VWF, and/or FVIII in VWD [12].

**Figure 2:**
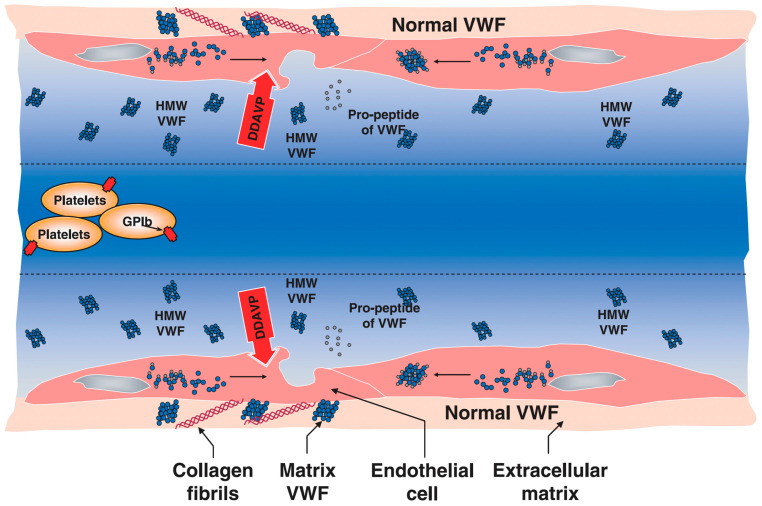
A schematic representation of the mechanism of action of DDAVP. Reproduced from Federici, 2008 [1]. Reproduced with the permission of the publisher.

DDAVP binds vasopressin V2 receptors on vascular endothelial cell membranes and activates cyclic adenosine monophosphate-mediated signalling, inducing VWF and FVIII release [1,13]. VWF is secreted as pre–pro-VWF, which is then cleaved by furin into native VWF and VWF pro-peptide [1] (Figure 2). The DDAVP effect on FVIII and VWF levels are dependent on the availability of stored FVIII/VWF, as well as the efficacy of secretion and clearance rate [14]. Large interindividual variability is observed in DDAVP responsiveness, limiting its universal use [12]. In particular, DDAVP is not used for treatment of severe VWD (VWF levels < 5 U/dL) or HA (FVIII levels < 1 U/dL). DDAVP also stimulates the release of tissue plasminogen activator, which is mostly complexed to alpha 2-plasmin inhibitor and does not degrade fibrin or fibrinogen [15].

The latest international (American Society for Hematology/International Society on Thrombosis and Haemostasis; ASH ISTH) 2021 guidelines for management of VWD recommends undertaking a DDAVP challenge and ongoing consideration for treatment, including with DDAVP, for all patients with VWF antigen (VWF:Ag) < 30 IU/mL who respond to DDAVP [8]. These guidelines also recommend a DDAVP trial in all patients with VWD who have VWF levels 30–50 IU/mL, or low VWF activity patients who otherwise do not have a contraindication to its use.

As a synthetic analogue, desmopressin can be given intravenously, subcutaneously, or intranasally, with monitoring of VWF and FVIII levels at baseline (pre-administration), and several time points post-administration. These may include at 30–60 min, 1.5–2 h, 4 h, and typically also 24 h post-administration to determine efficacy, with individual institutional practices varying. The dosing of intravenous administration is 0.3 mg/kg (maximum dosing 20–30 mg) in 50 mL of 0.9% sodium chloride and given over 20–30 min. Alternatively, subcutaneous administration is performed, typically also at 0.3 mg/kg. Intranasal spray is less readily available, depending on geographical jurisdiction, with recommended dosing of two 150 mg sprays for patients over 50 kg and one spray for patients under 50 kg. Notably, desmopressin has several listed side effects, including headache, tachycardia, flushing, hypotension, hyponatraemia, and rarer seizures and coronary events [2,16,17]. Therefore, initial DDAVP use needs to be carefully monitored by trained clinical personnel before being considered for standard use in any given patient. However, serious side effects are rare. Tachyphylaxis is a common occurrence after repeat dosing [17].

Throughout this review, we aim to describe current laboratory and clinical evidence of the use of DDAVP in VWD and HA, but also for platelet function defects, pregnancy, and uremia. Examples of its use from our local practice in VWD and HA will also be included to demonstrate its broad efficacy.

## 2. Evidence of Efficacy and Use

### 2.1. Clinical Evidence and Laboratory Assays

#### 2.1.1. Von Willebrand Disease

VWD is classified into three major types (1, 2, and 3), with type 2 further classified into four types (2A, 2B, 2N, and 2M) [13]. Type 1 VWD is a quantitative disorder representing a reduction in VWF production; however, the VWF that is produced is functionally normal. Type 3 VWD is the most severe form of VWD and where no VWF is produced. Type 2 VWD represents qualitative disorders in which the VWF produced is functionally defective. DDAVP has greatest clinical utility in mild type 1 VWD but can be useful for some type 2 VWD cases. DDAVP is only useful for short-term therapy, with VWF concentrates representing the main clinical therapy. Nevertheless, the use of DDAVP provides a VWF concentrate sparing strategy.

The core laboratory tests used to diagnose and monitor treatment efficacy in VWD involve the assessment of FVIII activity (%, U/dL or U/mL) and VWF protein level (Ag) and activity. The assessment of VWF activity requires a multitude of assays, depending on the clinical situation and the patient’s VWD type. The multiple tests include the assessment of VWF protein binding to factor VIII, collagen, and platelets [10,18,19]. Good response to DDAVP is most often defined as achieving at least 2 times the baseline VWF level and the ability to raise both VWF and FVIII levels to >50 U/dL (or >0.50 U/mL), although other definitions exist [8,12,20,21]. Target VWF and FVIII levels are also recommended by the ASH/ISTH guidelines, defined as both FVIII and VWF activity levels > 50 U/dL for at least 3 days following major surgery, and >50 U/dL to >150 U/dL for neuraxial anesthesia [8]. There is variation in target level and duration for minor procedures; however, levels generally > 50 U/dL are recommended.

The increasing recognition of the 1C subtype of VWD, characterized by a reduced VWF half-life, is also important to define, given treatment implications [22,23]. VWF or FVIII levels should be performed at serial intervals including 4 h after administration of desmopressin to identify 1C VWD, which is defined by rapid clearance [22].

DDAVP represents an appropriate adjunct treatment choice for most cases of type 1 VWD but is also useful in type 2 VWD and some cases of acquired von Willebrand syndrome (AVWS) [24,25,26]. In a multicenter international study consisting of 229 patients with VWD, 31 of whom had type 2 VWD, biological complete responses were demonstrated in 89%, defined by both VWF ristocetin cofactor (VWF:RCo; a marker of VWF binding to platelets) and FVIII:C > 50 U/dL, with 10% having partial response, defined by 3 fold increase and VWF:RCo or FVIII:C < 50 U/dL, and 1% with no response, defined by <3 fold increased and VWF:RCo or FVIII:C < 50 U/dL; there was a strong correlation found in patients with baseline VWF:Ag < 30 U/dL (Fisher’s exact = 0.001) [26]. Among patients with type 1 VWD, patients with C1130F and R1205H mutations showed accelerated clearance (*n* = 15). Subsequently, in a meta-analysis of in patients with VWD type 1 [12], VWF:Ag (OR, 1.055 per U/dL; 95% CI 1.016–1.096), VWF activity (several assays included; OR, 1.048 per U/dL; 95% CI, 1.008–1.090 (noting the majority of VWF activity assays comprised VWF:RCo but others were assessed with different platelet binding assays), and FVIII coagulant (FVIII:C) (OR, 1.023 per U/dL; 95% CI 1.002–1.045) were associated with a higher proportion of complete response, whereas age and VWF collagen binding (VWF:CB) assays were not [12]. In patients with type 2 VWD, there were no significant determinants of DDAVP response found.

In a retrospective review, Chandrakumaran et al. (2023) found that 84 of 94 (89.4%) cases of VWD over a 15-year period responded to DDAVP [27]. In regard to pre-surgical treatments over this period, 99 procedures used DDAVP and 86 used DDAVP ± tranexamic acid (an antifibrinolytic agent), whereby excessive bleeding occurred in only 4 of 31 major procedures and 2 of 55 minor procedures. When treated with a combination of DDAVP, tranexamic acid, and VWF concentrate (Biostate), only 1 of 10 major procedures and 1 of 3 minor procedures resulted in postoperative bleeding.

In a large Italian cohort, 147 patients with VWD were prospectively reviewed for management of bleeding over a 1-year period. A total of 40% were treated with DDAVP alone (23% type 1, 17% type 2). The remaining 60% were treated with VWF concentrates, 15% of whom were type 1 VWD [28]. Nolan (2000) found that of 91 children and adults with VWD, 90% achieved normalization of VWF:Ag, as well as ristocetin cofactor (VWF:RCo) and collagen binding (VWF:CB) activity post-administration of DDAVP [29].

Genetic mutations in VWD may also affect DDAVP responsiveness, particularly when considering implications of VWF folding, storage, secretion, and interaction with other proteins. Atiq et al. (2022) found that all patients with type 1 VWD patients without an evident VWF gene variant demonstrated desmopressin response; however, patients with type 1 and type 2 VWD who had a VWF gene mutation showed variable response rates of 64.3% and 31.3%, respectively [30]. It has previously been found that patients with VWD who have mutations in the VWF gene D′-D3 domains tend to have the relatively greatest responses to desmopressin, while most non-responsive patients have mutations in the VWF gene A1-A3 domains [20] []. However, the practical usefulness of the genotype is limited, and the responsiveness is best established using a test dose or DDAVP trial [23].

AVWS is similar to inherited VWD, in that there is reduced VWF protein and/or activity, but instead of being inherited, AVWS results from a variety of conditions, including malignant disorders, cardiac valvular disease, and left ventricular assist devices [31]. AVWS is rare, but otherwise similar to VWD regarding laboratory findings and clinical severity [1,32]. DDAVP has also been useful in select cases of AVWS. In a retrospective international registry of AVWS consisting of 186 patients, the majority of patients demonstrated responsiveness to desmopressin; however, rates were variable depending on the underlying cause of AVWS [33]. Additional therapeutic options exist in this cohort, given the variable mechanism for AVWS, including VWF/FVIII concentrates and intravenous immunoglobulin (IVIg). In a cohort of AVWS secondary to monoclonal gammopathy, DDAVP was given to manage bleeding and found to be limited by the short half-life of VWF/FVIII, which is removed from circulation by various mechanisms [25]. To summarize, although DDAVP is generally not considered in this population, it could be considered in select cases.

#### 2.1.2. Hemophilia A

The mainstay of treatment in hemophilia A is factor VIII replacement in either episodic or prophylactic treatment depending on severity (ISTH guideline for congenital hemophilia A and B) [34]. This is especially true for most surgical procedures and for long term use. However, DDAVP should be considered in select individuals and circumstances. In particular, this would apply for patients with mild HA and for select, typically mild surgical procedures or short-term need. Early use of DDAVP in mild HA suggested that it raised plasma FVIII activity to 2–3 times above baseline levels; however, effectiveness was absent in those with severe disease and unmeasurable FVIII activity [5,35]. Initial small cohort studies demonstrated similar efficacy of DDAVP compared with infusion of plasma-based FVIII concentrates in patients with mild HA who were undergoing surgical procedures and dental extractions [5,36,37,38,39]. Subsequent studies have demonstrated similar effects.

In a cohort of 62 boys with mild FVIII deficiency, 47% had at least a twofold increase in FVIII levels from baseline one hour after DDAVP administration, with a higher likelihood of response in those with higher baseline FVIII levels and with increasing age [38]. In another study including 20 patients with mild HA, 75% achieved normalized FVIII levels (>40 U/dL) [29]. Loomans et al. (2018) found that as many as 40% of their moderately affected HA patients reached plasma FVIII levels of at least 30 U/dL or more after DDAVP, and 15% of them attained levels as high as 50 U/dL or more [21]. Despite these changes in FVIII levels, the authors did not directly investigate whether or not these corresponded to a beneficial clinical effect on hemostasis. Romano et al. (2023) [40] described patient perspectives of 508 patients with mild and moderate HA and DDAVP use, whereby 131 of 164 adults and 19 of 23 children reported DDAVP as effective for the management of acute bleeding. On further interrogation of perceived effectiveness, it was stated to be effective to treat mucosal bleedings in 60 (29%) adults and 14 (61%) children, and effective to treat larger bleeding events in 96 (47%) adults and 12 (52%) children [40]. Intranasal administration was the modality of choice for 138 of 182 (76%) patients. Stoof et al. found that in moderate HA, 17.6% had a complete response and 29.4% had a partial response (total responders 47%, *n* = 17) to DDAVP administration at 1 hr; however, the proportion who had a complete response dropped to 0% by 3 h. Partial response was only seen in 9.1% at 6 h [41].

Laan et al. (2025) [12] performed a meta-analysis including 71 articles on the use of DDAVP in bleeding disorders. DDAVP response in HA carriers was 0.90 (CI, 0.68–0.98), mild HA was 0.54 (CI, 0.43–0.64), and moderate HA was only 0.16 (CI, 0.11–0.22), indicating a lower response rate in patients with lower baseline FVIII activity levels [12].

Acquired HA is a rare but life-threatening clinical syndrome characterized by acquired autoantibodies against FVIII epitopes, leading to accelerated clearance from plasma and/or neutralization of FVIII activity in plasma [42,43]. Treatment is generally multifaceted including control of bleeding with FVIII bypassing agents (FEIBA, recombinant activated factor VIIa or activated prothrombin complex concentrates [44,45,46]), immunosuppression [47,48], and treatment of the underlying trigger. In the EACH2 registry of acquired HA in Europe, 24% of patients received FVIII or DDAVP therapy but these patients had higher baseline FVIII levels, lower inhibitor titres, less severe bleeding, and more often received concurrent tranexamic acid [46]. They also found that FVIII concentrates and DDAVP demonstrated lower efficacy than bypassing agents. In a systemic review including a total of 37 patients from 15 reports, Franchini & Lippi (2010) found that DDAVP was used for non-life-threatening hemorrhages and to cover minor surgical, invasive, or dental procedures in acquired HA with low titre inhibitors [42].

#### 2.1.3. Platelet Function

The formation of a primary plug involves blood platelets and how they interact with VWF and endothelial surfaces in order to activate secondary hemostasis and enhance platelet adhesion to a damaged blood vessel wall [49,50,51,52]. There have been several studies analyzing the effect of DDAVP in various platelet function defects. A number of studies show normalization in Platelet Function Assay (PFA)-100 and activated partial thromboplastin time (aPTT) [25,53,54,55]; however, other reports demonstrate no effect on in vitro assays [56,57]. Patients with Glanzman thrombasthenia and afibrinogenemia have not been shown to respond to DDAVP [58,59,60]. Pfueller et al. (1987) showed an increase in plasma FVIII:C, VWF:Ag, and VWF:RCo activity as well as shortened bleeding time on two occasions in a patient with δ-Storage pool deficiency [58]. Cordova et al. (2005) reported a case series of 19 children with Hermansky–Pudlak syndrome and found resolution of abnormal bleeding time in only two cases (11%), demonstrating overall lack of efficacy [61].

Nolan et al. (2000) found that of 22 child and adult patients with platelet function disorders, 77% demonstrated normalization of bleeding time assay post-DDAVP administration [29]. Based on previously published data, there is usually a good response to DDAVP in the majority of patients with storage pool deficiency, unless there were severe deficiencies of platelet-δ-granules [62,63]. DDAVP normalized the bleeding time and/or platelet function test PFA-100 in most patients with congenital platelet dysfunction [2,53,64,65,66], as well as in patients with thrombocytopenia and otherwise normal platelet function, but response varies according to baseline platelet count [67,68]. DDAVP responsiveness is greater in patients with platelet counts >50 × 10^9^/L [2]. Despite various case series assessing in vitro laboratory coagulation assay improvement, it should be noted that there is a paucity of published data on clinical efficacy. There is also insufficient evidence for the use of DDAVP in bleeding in patients with bone marrow failure syndromes undergoing intensive chemotherapy or stem cell transplantation for hematological malignancies. DDAVP administration normalized PFA-100 abnormalities in patients taking aspirin or tidopidine but not ticagrelor [69,70].

In a systemic review by Desborough et al. (2017) reporting results from ten trials and a total of 596 participants, the use of DDAVP pre-cardiac surgery in patients with platelet dysfunction (defined as secondary to antiplatelet use or cardiopulmonary bypass) reduced red cell transfusion (−0.65 units; 95% CI −1.16 to −0.13 units), lowered blood loss (−253.93 mL; 95% CI, −408.01 to −99.85 mL), and had a lower risk of re-operation due to bleeding (4.0% compared with 10%, Peto OR, 0.39; 95% CI, 0.18–0.84) [16].

Swieringa et al. (2015) found that DDAVP use in 16 patients with postoperative bleeding after cardiothoracic surgery did not affect the coagulation profile or whole blood thromboelasticity, but did increase VWF:Ag from median 116 U/dL to 160 U/dL (IQR 126–187, *p* = 0.007) [49]. This was secondary to an increase in high molecular weight VWF multimers, as well as an increase in collagen-dependent thrombus formation and platelet phosphatidyl-serine exposure.

#### 2.1.4. Uremic Platelet Dysfunction

Although there is limited definitive evidence that uremia induces reduction in FVIII and/or VWF, prolonged bleeding times are observed [2]. Despite this, the prolonged bleeding time seen in uremic patients has been shown to normalize in 75% of patients following intravenous infusion of DDAVP [71], as well as following subcutaneous and intranasal administration [72,73]. However, in subsequent studies of uremic patients undergoing low bleeding risk procedures, there has been no significant difference demonstrated in terms of bleeding events and transfusion requirements comparing DDAVP to placebo [74,75]. A retrospective case series also demonstrated that DDAVP was unable to control bleeding in patients with uremia who had gastrointestinal bleeding [76]. There is some evidence that EPO agonists and conjugated estrogens in end stage renal failure associated uremia help in correcting hemostatic abnormalities in these patients [77].

#### 2.1.5. Liver Cirrhosis

Chronic liver disease and cirrhosis often leads to abnormal in vitro coagulation tests and may present clinically as either pro-thrombotic or pro-hemorrhagic phenotypes [78,79]. DDAVP has been used in a number of reports in liver disease to reduce bleeding in those with a bleeding phenotype [80]. Burroughs et al. (1985) found that 15 patients with liver cirrhosis who received 0.3 mg/kg of DDAVP had significantly shortened in vitro bleeding time as well as improvements in APTT, FVIII activity, and VWF:Ag and VWF:RCo activity [81]. In a later study, Lopez et al. (1997) found similar in vitro results in 14 patients with liver cirrhosis following 0.3 mg/kg of DDAVP but also found a reduction in mean arterial blood pressure (12 ± 8%, *p* < 0.05) and sustained shortening in bleeding time at 6 and 24 h following administration [82]. de Franchis et al. (1993) attempted to demonstrate clinical efficacy of DDAVP in combination with terlipressin in cirrhotic patients with active variceal bleeding and found that patients who received both treatments had higher rates of recurrent bleeding within 24 h (13 of 24 patients) compared with those who received terlipressin alone (6 of 22 patients) [83]. Interestingly, Arshad et al. (2015) found that in 10 patients with cirrhosis, DDAVP administration did not result in changes to high molecular weight VWF multimers, VWF:Ag, and VWF-dependent platelet adhesion, but did find similar increases in FVIII activity and VWF pro-peptides in patients with cirrhosis compared with patients with mild HA [84]. These results may explain the lack of published clinical benefit in bleeding patients with cirrhosis to date.

#### 2.1.6. Pregnancy

Pregnancy and delivery represent major hemostatic challenges. Post-partum hemorrhage can represent a unique challenge in women with inherited bleeding disorders. DDAVP use has been limited due to concerns for fetal and maternal safety. Ray (1998) evaluated DDAVP use in pregnant women with diabetes insipidus and found no DDAVP-related maternal or neonatal adverse events [85]. In a systemic review by Al Arashi et al., DDAVP use in pregnancy appears safe with the only safety concern being 1% risk of hyponatremia in 273 pregnancies (*n* = 2) and low APGAR score in 0.5% (*n* = 1) [86]. This study included a mixed cohort, with VWD being the largest group, with 212 (78%) pregnancies, followed by HA carriership (31, 11%), platelet function disorder (14, 5%), Hermansky–Pudlak syndrome (8, 3%), FXI deficiency (3, 1%), Ehlers Danlos syndrome (2, 1%), and a combination of disorders (2 pregnancies, 1%).

### 2.2. Illustrative Case Examples

The following section provides some illustrative case examples of DDAVP trial data from select patients with HA or VWD. DDAVP trials in these patients were performed to assess the clinical utility of DDAVP as a potential adjunct therapy for these patients. In addition, DDAVP data can also be used to firm up a diagnosis of VWD in a patient with an unclear VWD subtype [87,88].

#### 2.2.1. VWD

**Figure 3:**
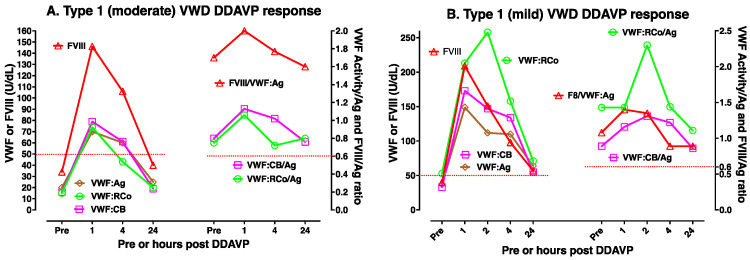
Two case examples of DDAVP use in type 1 VWD for a moderately (**A**) and for a mildly (**B**) affected patient. Different patients will yield different test patterns or responses to DDAVP, highlighting the need for DDAVP trials such as shown here to establish responsiveness in any individual patient. In both these examples, FVIII and VWF levels have all been raised to above 50 U/dL for both patients post-DDAVP but were maintained above 50 U/dL only for the mildly affected patient. In both patients, the ratio of FVIII to VWF:Ag (FVIII/VWF:Ag) and VWF activity/VWF:Ag ratios were normal (>0.6) at all time points (i.e., pre- and post-DDAVP).

**Figure 4:**
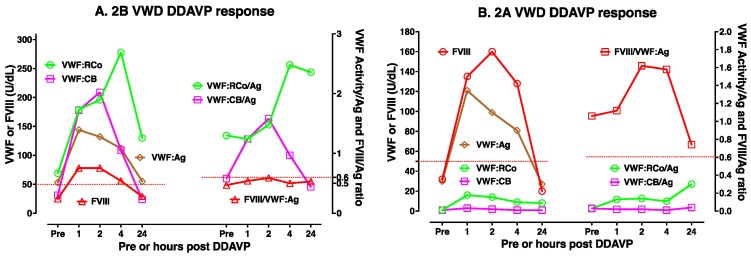
An example of DDAVP use in type 2B VWD (**A**) and type 2A VWD (**B**). Different patients will yield different test patterns or responses to DDAVP, highlighting the need for DDAVP trials such as shown here to establish responsiveness in any individual patient. DDAVP use in type 2B VWD is usually considered contra-indicated but may be useful in select patients (notably those without thrombocytopenia). In the type 2B patient shown (**A**), DDAVP use raised all VWF parameters and FVIII to above 50 U/dL for 4 h, and VWF activity/Ag ratios normalized (i.e., >0.6) and did not lead to thrombocytopenia. In this type 2B VWD patient, however, assessed FVIII levels were unusually low. For the 2A VWD patient, VWF:Ag and FVIII levels both increased above 50 U/dL for 4 h, but VWF activity levels did not, and all VWF activity/Ag ratios were <0.6 at all time points. Thus, this 2A VWD patient was DDAVP responsive for FVIII but not for VWF activity. In summary, DDAVP use showed only partial clinical utility for these patients.

**Figure 5:**
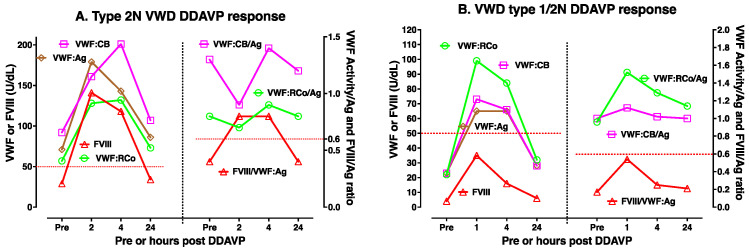
An example of DDAVP use in type 2N VWD (**A**) and combined type 1/type 2N VWD (**B**). Different patients will yield different test patterns or responses to DDAVP, highlighting the need for DDAVP trials such as shown here to establish responsiveness in any individual patient. DDAVP use in type 2N VWD may or may not produce a sufficient DDAVP response, in particular for FVIII. In the first type 2N patient shown (**A**), DDAVP use raised all VWF parameters and FVIII to above 50 U/dL for 4 h, and the ratio of FVIII to VWF:Ag (FVIII/VWF:Ag) normalized (i.e., >0.6) for this time period. In the second type 2N VWD, where a type 1 VWD was also concomitantly present, neither FVIII nor the ratio of FVIII to VWF:Ag (FVIII/VWF:Ag) normalized; however, VWF activity levels (VWF:RCo and VWF:CB) both normalized. In summary, DDAVP use showed only partial clinical utility for both patients.

**Figure 6:**
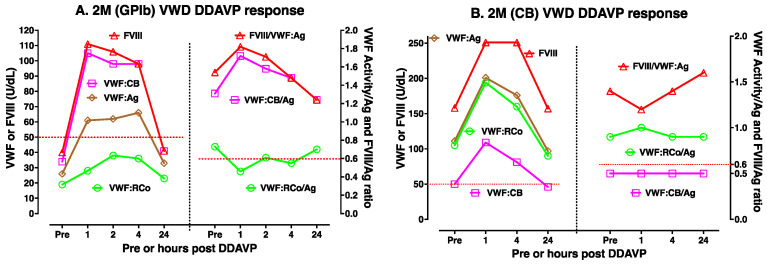
Examples of DDAVP use in type 2M VWD. Different patients will yield different test patterns or responses to DDAVP, highlighting the need for DDAVP trials such as shown here to establish responsiveness in any individual patient. (**A**) DDAVP use in type 2M VWD expressing a glycoprotein Ib (GPIb) binding defect manifesting as relatively reduced VWF:RCo activity. In this patient, FVIII, VWF:Ag, and VWF:CB all normalized to >50 U/dL for at least 4 h, but VWF:RCo did not. Also, the ratio of FVIII to VWF:Ag (FVIII/VWF:Ag) and the VWF:CB/Ag ratios remained normal (>0.6) at all time points, whilst the VWF:RCo/Ag ratio did not. (**B**) DDAVP use in type 2M VWD expressed a collagen binding (CB) defect manifesting as relatively reduced VWF:CB activity. In this patient, FVIII, VWF:Ag, and VWF:RCo remained normal (i.e., >50 U/dL) at all time points, but VWF:CB did not. Also, the ratio of FVIII to VWF:Ag (FVIII/VWF:Ag) and the VWF:RCo/Ag ratios remained normal (>0.6) at all time points, whilst VWF:CB/Ag ratio did not. In summary, DDAVP use showed only partial clinical utility for both patients but also aided in confirming their respective phenotypes as 2M VWD of a GPIb type (**A**) or of a CB type (**B**).

DDAVP use is most useful in type 1 VWD, especially where baseline VWF levels are highest. Figure 3A,B shows two examples of DDAVP use in moderate/mild type 1 VWD. DDAVP use is considered contraindicated in type 2B VWD, since there is some fear that this may lead to the release of abnormal VWF that then acts to clear platelets and worsen thrombocytopenia. However, DDAVP use is associated with improving VWF level and activity in some type 2B VWD patients, in particular those without baseline thrombocytopenia (case example shown in Figure 4A). DDAVP will also raise FVIII levels and VWF:Ag levels (but not VWF activity) in type 2A VWD (case example shown in Figure 4B); accordingly, the release of VWF with abnormal activity is expected to be less clinically efficacious in these patients. DDAVP will also raise VWF levels and FVIII levels in 2N VWD, although levels of FVIII may fall relatively quickly due to clearance (two case example shown in Figure 5A,B). Finally, the effect of DDAVP in 2M VWD depends on the genetic defect and baseline VWF tests. Thus, 2M VWD cases expressing a platelet binding defect will show a rise in VWF:CB activity but not platelet binding activity (case example shown in Figure 6A), whereas 2M VWD cases expressing a collagen binding defect will show a rise in platelet binding activity but not VWF:CB activity (case example shown in Figure 6B).

#### 2.2.2. Hemophilia A

**Figure 7:**
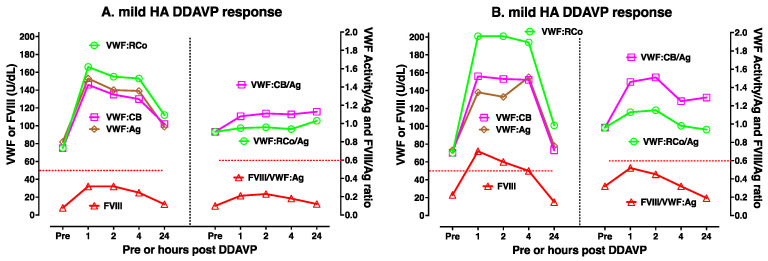
Examples of DDAVP use in mild hemophilia A (HA). Different patients will yield different test patterns or responses to DDAVP, highlighting the need for DDAVP trials such as shown here to establish responsiveness in any individual patient. (**A**): DDAVP use in mild HA with baseline FVIII:C < 10 U/dL. In this patient, all test parameters rose after DDAVP, but despite 2–3-fold increases, FVIII did not normalize to >50 U/dL at any time point. (**B**): DDAVP use in mild HA with baseline FVIII:C ~20 U/dL. In this patient, all test parameters rose after DDAVP, and FVIII did normalize to >50 U/dL for at least 4 h. In summary, then, DDAVP would be of greater clinical utility for the second mild HA patient (**B**) than the first mild HA patient (**A**). Note that the higher the baseline FVIII level, the more likely that FVIII levels will normalize post-DDAVP. However, the higher the baseline VWF level, the greater the risk that VWF levels may rise to >200 U/dL.

As noted previously, efficacy of DDAVP in HA also depends on initial baseline FVIII levels (Figure 7A,B). Also, caution is indicated in DDAVP use for HA whereby VWF levels will also rise, perhaps to high levels that may pose a risk factor for thrombosis.

### 2.3. Precautions and Contraindications

As mentioned, DDAVP is reportedly contraindicated in type 2B VWD due to new or potentially worsening thrombocytopenia that may arise from increased platelet binding [8]. However, we and others have used DDAVP safely in a cohort of type 2B VWD cases, notably variants that do not express thrombocytopenia or who do not show worsening of platelet count with DDAVP use. Of additional note, DDAVP lacks efficacy in type 3 VWD due to the absence of endogenous VWF stores. DDAVP should not be used in patients with active cardiovascular disease or patients with seizures, due to the potential risk of vasospasm and hyponatraemia, respectively, nor in patients <2 years of age or patients with type 1C VWD in the context of surgery [8,^89^]. Notably desmopressin has been used safely in women who are pregnant but should be avoided in women with pre-eclampsia or those with cardiovascular disease [85,89,90]. However, despite clinical utility in a cohort of patients, DDAVP use only leads to short-term release of FVIII and VWF and thus is only affective for short-term therapy and for minor surgical procedures. In most cases of HA and VWD, the primary therapy will be FVIII and VWF concentrates, respectively, and thus DDAVP is best considered as an adjunct FVIII and VWF concentrate sparing therapy.

## 3. Challenges and Future Directions

Despite its established role in mild HA and VWD, the therapeutic potential of DDAVP remains underexplored. Research is needed to demonstrate clinical efficacy in larger cohorts in patients receiving antiplatelet therapy, liver disease, or congenital or acquired bone marrow failure. The role of DDAVP in perioperative hemostasis for patients with borderline coagulation profiles or thrombocytopenia may also warrant further study. Further research is needed in pediatric and geriatric populations from tailored dosing strategies to optimize safety and efficacy. Endothelial function assays and semi-quantitative VWF multimer assays may refine patient selection and response prediction. The potential efficacy of DDAVP in combination with antifibrinolytics or novel hemostatic agents may represent an avenue for future investigation in patients with complex bleeding phenotypes.

## 4. Conclusions

DDAVP is a readily available and widely used non-blood product in patients with HA, VWD, and platelet function disorders, with evidence of laboratory and clinical reduction in bleeding, and growing evidence in perioperative management, pregnancy, and uremia. Nevertheless, for most cases of HA and VWD, the primary therapy will be FVIII and VWF concentrates, respectively. Further research is also needed, in particular to demonstrate clinical benefit in less well explored patient or disease populations.

## Data Availability

No new data were created in this review. All data provided is available in the public arena, except for the DDAVP examples, which represent cases from our institution, used here only for educational purposes.

## Abbreviations

The following abbreviations are used in this manuscript:

APTT	Activated Partial Thromboplastin Time
ASH	American Society for Hematology
AVWS	Acquired von Willebrand Syndrome
DDAVP	1-deamino-8-D-arginine, or desmopressin
FVIII	Factor VIII
HA	Hemophilia A
ISTH	International Society on Thrombosis and Haemostasis
VWD	Von Willebrand Disease
VWF	Von Willebrand Factor
